# Short-term functional outcome and pain assessment after spinal decompression for spondylodiscitis

**DOI:** 10.25122/jml-2026-0014

**Published:** 2026-04

**Authors:** Bogdan Șendrea, Bogdan Gabriel Voicu, Sebastian Mihai Valeanu, Andrei Tudorache, Mihaela Gabriela Stanciu, Romica Cergan

**Affiliations:** 1 Foisor Clinical Hospital of Orthopedics, Traumatology and Osteoarticular Tuberculosis, Bucharest, Romania; 2 Carol Davila University of Medicine and Pharmacy, Bucharest, Romania

**Keywords:** Frankel paraplegia grade, visual analog scale, Oswestry Disability Index, posterior spinal decompression, spondylodiscitis, spinal tuberculosis

## Abstract

Spondylodiscitis, though rare, is a serious condition that can lead to disability or death. It may be caused by pyogenic, granulomatous (e.g., tuberculosis, brucellosis, fungal), or rarely, parasitic infections. Pain and neurological deficits typically result from bone destruction, deformity, and mass effect. Various microbiological agents have been identified in the literature, with more frequent spinal infections caused by pyogenic bacteria, such as *Staphylococcus aureus*, as well as by less common agents, including *Mycobacterium tuberculosis* and fungi, such as *Candida albicans*. Between February 2013 and May 2020, patients underwent posterior decompression surgery with specimen collection for microbiological analysis, transpedicular instrumental fixation, and deformity correction. Inclusion criteria were spinal pain, neurological deficit, spondylodiscitis with radiological or MRI confirmation, and positive microbiological results. Patients under 18, those requiring multiple surgeries, or those with degenerative or conservatively managed cases were excluded. Post-surgical treatment included 6 weeks of dual-antibiotic therapy for pyogenic infections, 1 year of antituberculous therapy with four drugs for the first 3 months, and 6 months of antifungal therapy, consisting of 4 weeks of intravenous treatment followed by oral therapy. Descriptive statistical methods were used in this study. Of the 67 patients, 73.13% had lumbar involvement. Thirty underwent single-level fixation; 37 had multi-level fixation. Pain scores (VAS) improved consistently at 6 weeks and 3 months post-op. All patients initially had neurological deficits, with functional improvement shown by better Oswestry Disability Index scores and Frankel grades after surgery. Surgical intervention was effective in relieving pain, correcting deformities, and improving function in spondylodiscitis. Obtaining a microbiological diagnosis during decompression is crucial for guiding targeted therapy and minimizing antibiotic resistance.

## Introduction

Spondylodiscitis, while relatively uncommon, represents a clinically significant condition associated with substantial morbidity and potential mortality [1]. Spinal infections may be classified etiologically into pyogenic and granulomatous forms, the latter encompassing tuberculous, brucellar, and fungal causes; parasitic causes are exceedingly rare [2]. More aggressive pyogenic spinal infections include several related entities. Spondylodiscitis represents a spectrum of disease involving varying degrees of vertebral osteomyelitis, spondylitis, and discitis, which are regarded as different manifestations of the same pathological process. An epidural abscess may occur either as a primary condition or as a complication of spondylodiscitis. In rare cases, facet joint involvement (arthropathy) may also be observed. Bone stock destruction with subsequent deformity and mass effect from the infectious process are the main factors involved in pain and neurological deficit progression. Various microbiological agents have been identified in the literature, with more frequent spinal infections arising from pyogenic bacteria such as *Staphylococcus aureus*, to the less common *Mycobacterium tuberculosis*, and even fungi such as *Candida albicans* [1-4]. Infection pathways are divided into hematogenous (most common), iatrogenic, or via continuity from other infectious processes (such as pyelonephritis or pulmonary empyema). Regardless of the microbiological agent involved in the infectious process, the functional disability secondary to disease progression bears a heavy burden for the patient and also the healthcare system, which is deemed to carry out treatment. In this paper, the clinical and functional outcomes were assessed before and after spinal surgical debridement and posterior fusion with transpedicular instrumentation (Visual Analog Scale for pain, Frankel Paraplegia Scale, and Oswestry Disability Index) [5].

This study aimed to evaluate the clinical and functional outcomes following posterior decompression and transpedicular instrumentation in patients with spondylodiscitis.

## Material and Methods

This was a retrospective single-center cohort study conducted at the Foisor Clinical Hospital for Orthopedics, Traumatology, and Osteoarticular Tuberculosis in Bucharest, Romania. We studied 67 patients with spondylodiscitis. All patients underwent open posterior access to the medullary elements for decompression, with specimen collection for microbiological analysis, transpedicular fixation, correction of secondary deformity when necessary, and fusion between February 2013 and May 2020. The inclusion criteria were as follows: all patients presented with neurological deficit, spinal pain, and evidence of vertebral involvement on imaging, either radiography or magnetic resonance imaging (MRI). All cases required microbiological confirmation, via positive cultures or equivalent tests, to establish spondylodiscitis as the cause of spinal neurological symptomatology. Laboratory findings were also recorded, although a small percentage of patients presented with normal inflammatory markers. Some patients had received prior empirical antibiotic treatment, which increased the difficulty of microbiological identification.

Values for the Visual Analogue Scale (VAS) for pain, ranging from 0, indicating no pain, to 10, indicating the worst pain ever encountered; Frankel paraplegia grade, ranging from grade E, indicating no neurological symptoms, to grade A, indicating complete motor and sensory loss; and the Oswestry Disability Index, ranging from 0, indicating no disability, to 50, indicating complete disability, were recorded before surgery and at subsequent 6-week intervals up to 3 months after surgical intervention.

Exclusion criteria comprised the need for multiple spinal surgical interventions; age under 18 years, as pediatric patients in Romania are managed in separate pediatric orthopedic departments; confirmed degenerative spinal pathology without microbiological confirmation; and favorable clinical outcomes with conservative management, with or without antibiotic therapy.

All patients underwent posterior surgical decompression with transpedicular instrumentation and microbiological identification, either by staining or culture. Appropriate antimicrobial therapy was implemented according to the identified pathogen: 6 weeks of dual-antibiotic therapy for pyogenic bacterial infections; 1 year of antituberculous therapy for tuberculous infections, with four antituberculous agents administered during the first 3 months; and, for fungal infection, an initial 4 weeks of intravenous therapy followed by continued antifungal treatment for up to 6 months.

For patients with low tolerance to pain during orthostatic activities, rigid orthoses were formally recommended upon discharge.

## Results

**Figure 1:**
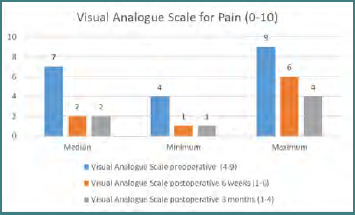
Visual Analog Scale for Pain: preoperative and at 6 weeks and 3 months post-surgery

Regarding the Visual Analog Scale (VAS) values recorded before surgery and at the 6-week and 3-month postoperative intervals, there was a consistent decrease in reported pain scores, as evidenced by the minimum, maximum, and median values for the group. However, the decrease was less evident between the postoperative follow-up intervals, suggesting that maximal pain reduction occurred by 6 weeks (Figure 1).

Out of the 67 patients, 49 (73.13%) had lumbar spine involvement. Thoracic spine involvement was identified in 43 patients (64.17%) (Table 1). Thirty patients (44.77%) presented with single-segment infection, 36 patients (53.73%) had infection involving two spinal segments, and one patient had infection spanning three spinal segments. Regarding fixation criteria, 30 patients (44.77%) underwent single-segment spinal fixation, including 18 patients (26.86%) in the thoracic segment and 12 patients (17.91%) in the lumbar segment. The remaining 37 patients (55.22%) required multi-segment spinal fixation (Table 2).

**Table 1:** Spinal segment involvement in the population

Spinal segment involvement	No. of patients	Percentage of total
Thoracic	43	64.17%
Lumbar	49	73.13%
Sacral	13	19.40%
		
1 segment	30	44.77%
2 segments	36	53.73%
3 segments	1	1.49%

**Table 2:** Spinal segment fixation in the population

Spinal segment fixation	No. of patients
Lumbar	12
Lombo-sacral	12
Thoracic	18
Thoraco-lombo-sacral	1
Thoraco-lumbar	24

**Figure 2:**
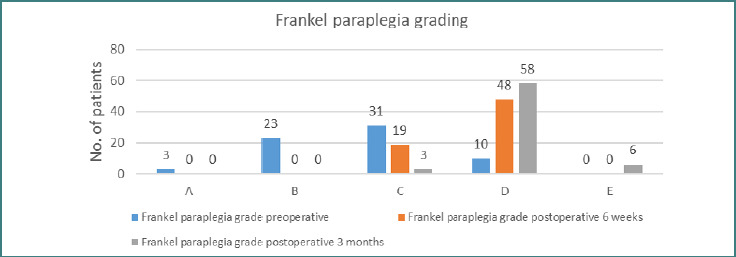
Frankel paraplegia grading records: preoperative, 6-week, and 3-month follow-up

Regarding Frankel paraplegia grading (Figure 2), all patients presented with neurological impairment before surgery. The impairment ranged from grade D (indicating incomplete motor loss) in 10 patients to grade A (indicating complete motor and sensory loss) in three patients. After surgery, overall neurological improvement was observed in all patients at both the 6-week and 3-month intervals, with further improvement noted between postoperative assessments. By the 3-month follow-up, most patients (58 in total) were classified as grade D, six as grade E (indicating no neurological deficit), and only three remained in grade C (indicating nonfunctional motor strength with preserved sensitivity).

**Figure 3:**
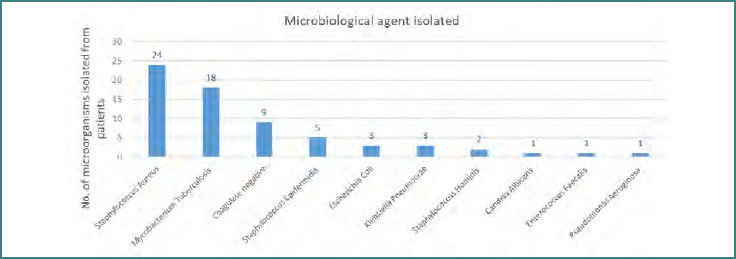
Microbiological agents isolated in patients with spondylodiscitis

Regarding the Oswestry Disability Index values recorded during the study, all patients showed decreased scores, reflecting functional improvement (Figure 3). The maximum preoperative score of 40 points decreased to 32 points postoperatively and to 10 points at follow-up. The minimum preoperative score of 15 points decreased to 5 points postoperatively and remained stable thereafter. The median score also decreased after surgery, from 30 points preoperatively to 20 points at initial follow-up and then to 7.5 points at later follow-up.

**Figure 4:**
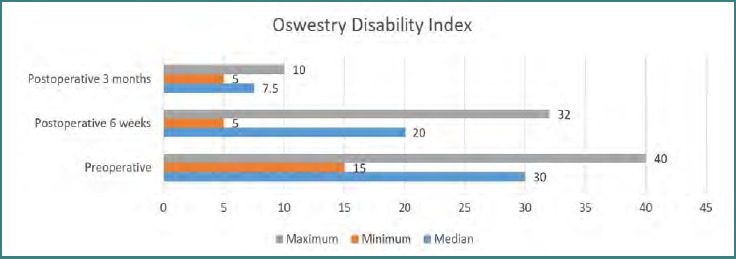
Oswestry Disability Index values recorded before and after surgery (possible score between 0 and 50)

**Figure 5:**
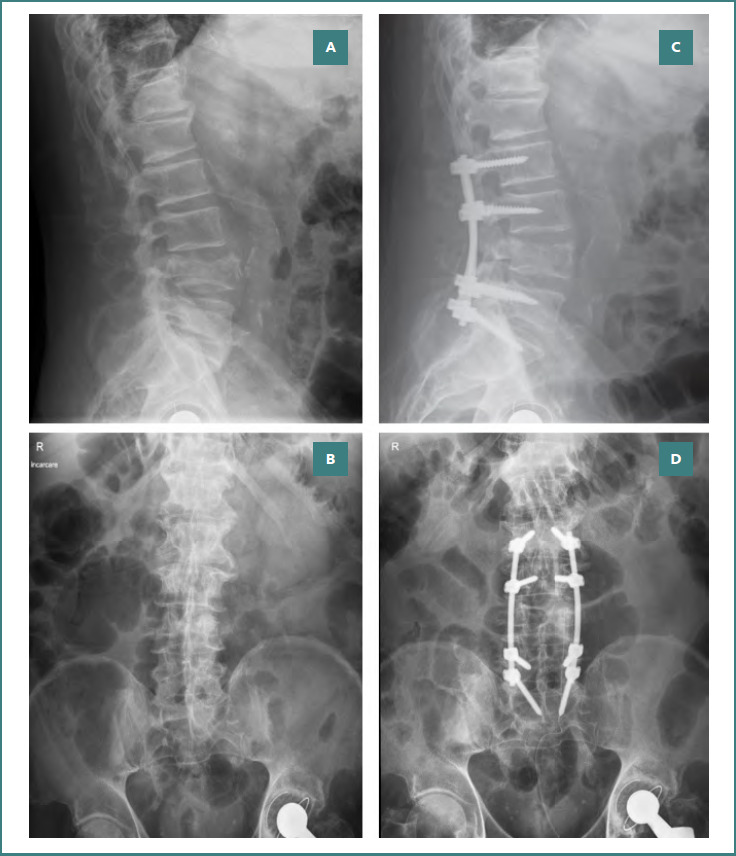
Preoperative and postoperative radiographs of a patient with L3–L4 spondylodiscitis treated by posterior decompression and L2–S1 fusion with pedicle screw instrumentation. A, **Preoperative sagital view;** B, **Preoperative anteroposterior view;** C, **Postoperative sagital view;** D, **Postoperative anteroposterior view**.

The microbiological agents responsible for spondylodiscitis were also quantified (Figure 4). The relatively high proportion of patients with mycobacterial infection, observed in 18 cases (26.86%), is consistent with the known epidemiological context, as Romania has historically reported one of the highest tuberculosis incidences in the European Union, with recent estimates of approximately 50–60 cases per 100,000 population, substantially above the EU average [6]. This sustained burden of tuberculosis, including extrapulmonary forms, may contribute to the observed frequency of mycobacterial spinal infections in the studied cohort. The single case of fungal infection warrants consideration of routine fungal screening in patients with spondylodiscitis. Although rare, fungal infection should still be considered. Radiological imaging from one case, before and after posterior decompression and pedicle fixation, is shown in Figure 5A-D.

## Discussion

All patients included in the study met neurological criteria warranting further workup, including laboratory and imaging studies. After completion of paraclinical testing, the cases were reviewed, and a therapeutic regimen was initiated, consisting of surgical treatment in this study. Clinical decision-making was not influenced by the study design, as the analysis was performed retrospectively.

Most cases of early-stage pyogenic vertebral osteomyelitis respond well to conservative treatment. In cases of pyogenic vertebral osteomyelitis refractory to conservative treatment, operative treatment is warranted. A thorough and radical debridement of all infected or necrotic tissue is mandatory, and appropriate antibiotics are required. The use of metallic implants in an infected area of the spine is safe and does not lead to persistent or recurrent infection [7].

The 2015 Infectious Diseases Society of America clinical practice guidelines recommend raising suspicion in patients with new or worsening back or neck pain accompanied by fever and elevated erythrocyte sedimentation rate (ESR) or C-reactive protein (CRP) levels. Bacterial blood cultures, both aerobic and anaerobic, should be obtained in two sets, particularly in the context of prior bloodstream infection or infective endocarditis, fever, and new neurological symptoms, with or without back pain. Patients should undergo spinal magnetic resonance imaging (MRI). When an MRI cannot be obtained, computed tomography (CT) or positron emission tomography (PET) may be considered. Fungal blood cultures should be obtained when at risk for fungal infection. An interferon-γ release assay should be obtained in pauci-symptomatic patients or in those at risk for *Mycobacterium tuberculosis* infection (i.e., originating from or residing in endemic regions or with risk factors). Finally, evaluation by an infectious disease specialist and a spine surgeon should be considered [8].

In patients with improvement of clinical symptoms despite worsening bony imaging findings, surgery should be temporized. Surgical intervention (whether sole decompression through either anterior or posterior or combined approaches or with associated instrumentation techniques) is warranted in patients with progressive neurologic deficits, recurrent bloodstream infection, progressive deformity, and spinal instability with or without pain despite adequate antimicrobial therapy. The goal of surgery, apart from microbiological debulking of the peri-dural space abscess and microbiological agent acquisition, should be to pursue the relief of compression of the spinal cord, cauda equina, or nerve roots and spinal stabilization to prevent further damage to the neural elements [7].

Valancius *et al*. reviewed the management strategies of 196 patients with spondylodiscitis treated at a single institution over 10 years. Of these, 100 patients required surgical intervention. In the study group, 46 patients presented with neurological deficit, three patients presented with cauda equina syndrome, and 10 patients presented with paraplegia.

Four surgical strategies were used. Posterior debridement with pedicle screw instrumentation was performed in 75 patients, while 19 patients underwent posterior debridement without instrumentation. Anterior debridement alone was performed in seven patients, and combined anteroposterior debridement with posterior pedicle screw instrumentation was performed in 16 cases. Overall, 12 patients had mild neurological impairment, four patients had paraplegia, and 27 patients had chronic residual pain of varying degrees [9].

Although systemic inflammatory markers are routinely used to identify patients at greater risk of treatment failure, their levels may paradoxically increase during the first few weeks after diagnosis and treatment, despite clinical improvement [10].

Patients with persistent or progressive pain, systemic symptoms of infection, undrained or partially drained large epidural abscess, or persistently elevated systemic inflammatory markers may be at highest risk for treatment failure [11].

The need for metallic implant instrumentation remains a clinical controversy among many authors. Certain surgeons argue that a metallic foreign body may interfere with successful eradication of infection. Many surgeons hesitate to place the implant directly in contact with the infection site after debridement because the implant surface may serve as a bacterial attachment site, facilitating biofilm development and microbial persistence [12,13].

Although not always mandatory, spinal instrumentation may sometimes be unavoidable, especially in cases of gross spinal deformity, instability, and neurological compression secondary to large bone stock defects. To prevent biofilm formation, adequate antibiotic treatment must be instituted, further underscoring the need to identify the causative microorganism. Antibiotic stewardship principles should be implemented whenever possible to avoid treatment failure and the selection of multidrug-resistant microorganisms [14].

As for neurological recovery, the study results are consistent with other literature. Patidar *et al*. found no post-surgical neurological deterioration in a study of patients who underwent a posterior approach debridement and pedicle instrumentation. The authors observed favorable outcomes in all cases, although similarly, not all patients attained full neurologic recovery [15].

The retrospective design represents an inherent limitation of this study. Additionally, the lack of uniformly available laboratory parameters—such as white blood cell counts, erythrocyte sedimentation rate, and C-reactive protein levels—as well as inconsistencies in imaging studies, limited the robustness of the statistical analysis, as many patients presented with paraclinical investigations performed at different institutions. Another important limitation is the absence of a control group undergoing conservative (non-surgical) management, which restricts comparative outcome assessment. Furthermore, follow-up was limited to a relatively short duration (3 months), and extended long-term evaluation was not feasible due to insufficient available data [16,17].

## Conclusion

In our cohort, posterior decompression and instrumentation were associated with significant short-term clinical and functional improvement, including deformity correction, relief of neural compromise, and restoration of function. Therefore, this approach should be pursued whenever poor neurological evolution is present in patients with highly suspected or confirmed spondylodiscitis.

Spondylodiscitis remains rare; however, because of its potentially life-threatening complications, it should always be included in the differential diagnosis of axial skeletal pain. A microbiological diagnosis is essential and can be obtained during posterior decompression, allowing appropriate selection of therapeutic agents and helping to avoid the potential development of multidrug-resistant bacteria.
